# Open-source modular resistance welding equipment for thermoplastic composites

**DOI:** 10.1016/j.ohx.2026.e00839

**Published:** 2026-09-12

**Authors:** Jonas Frank Reis, Luís Felipe Barbosa Marques, Miguel Omena Lucas Vieira, Davi Nascimento Gomes, Thiago Alves Kotz, Luis Rogerio de Oliveira Hein

**Affiliations:** Department of Materials and Technology, School of Engineering and Science, São Paulo State University (UNESP), Guaratinguetá, State of São Paulo, Brazil

**Keywords:** Resistance welding, Thermoplastic composites, Welding stations, Process monitoring

## Abstract

This paper presents an integrated open-source platform for resistance welding of thermoplastic composites, developed as a low-cost, self-contained alternative to expensive industrial welding stations. The system combines automated pressure control, real-time instrumentation, and automated data logging within a modular hardware architecture consisting of an aluminum frame and an electrically insulating wooden base that minimizes current leakage, complemented during operation by external ceramic insulator plates, not a permanent component of the equipment, positioned at the sample-electrode interface, where they act as the thermal barrier at the joint and protect the wooden structure from direct exposure to weld interface temperatures. Pressure is regulated through a closed-loop feedback system using an Arduino-controlled linear actuator and load cell. A dedicated Python-based graphical user interface provides high-fidelity process control and monitoring, featuring real-time visualization of voltage versus time, continuous acquisition of temperature (Type-K thermocouple), voltage, current, and power, and on-the-fly calculation of power density and heating element resistance. Power is supplied by a Huawei R4850G2 rectifier and JUNTEK DPM8624 converter, fully integrated into the control logic. At the end of each welding cycle, the software automatically generates a comprehensive PDF report (including process parameters, specimen dimensions, and the voltage–time graph) and a synchronized raw data log file (.txt) containing voltage, current, power, pressure, and temperature sampled every second. The platform enables precise, repeatable weld quality and accessible data acquisition for research and small-scale manufacturing of thermoplastic composite joints.

## Graphical abstract

**Figure:**
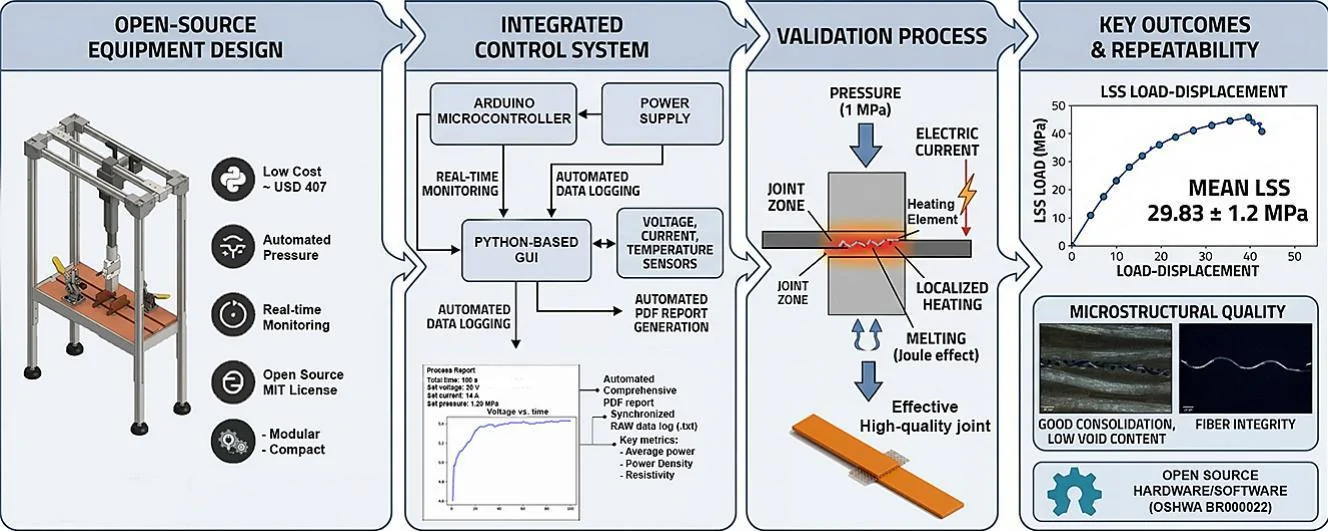

Specifications table

**Table:** 

Hardware name	*OpenWelT (Open source Welding for Thermoplastics)*
Subject area	• Engineering and materials science • Educational tools and open source alternatives to existing infrastructure • General
Hardware type	• Field measurements and sensors • Electrical engineering and computer science • Mechanical engineering and materials science
Closest commercial analog	*No commercial analog is available.*
Open source license	*Hardware: CERN-OHL-S v2.0* *Software: MIT LicenseDocumentation: CC BY 4.0*
Cost of hardware	*USD 159.83 – Structure part* *USD 247.36 − Electric components* *USD 407.19 – Total equipment cost*
Source file repository	http://doi.org/10.17632/8tb37yjp9m.3
OSHWA certification UID	*BR000022*

## 1 Hardware in context

The increasing use of advanced polymer matrix composites in lightweight structures is mainly due to their higher specific strength, and greater resistance to corrosion and fatigue than most metals and alloys applied in structures, becoming pronounced in the aerospace, automotive, and wind energy sectors [1], [2], [3], [4]. The aerospace sector heavily favors fiber-reinforced thermoplastic composites for design and manufacturing, as these materials offer a 20% to 30% savings in overall weight alongside a roughly 25% decrease in total production costs [5], [6], [7]. Although thermoset composites are used in most applications, interest in developing thermoplastic composites is driven by their increased damage tolerance [8], [9], excellent vibration damping [10], high impact resistance [11], [12], [13], [14], [15], fracture toughness [16], [17], short processing cycles [18], recyclability and reformability [19], [20], [21].

Furthermore, another factor in the growing use of thermoplastic composites in industries, especially aerospace, is their weldability [3]. This is due to the intrinsic property of thermoplastic matrices, which can be melted when heated [22]. Although melting causes a reduction in molecular weight, thermoplastics retain their original mechanical properties after cooling [23].

The welding of thermoplastic composites requires heating the joint interface above the polymer's glass transition temperature (for amorphous polymers) or melting temperature (for semi-crystalline ones) so that the polymer chains can diffuse across the interface. The joint then consolidates under pressure during cooling. Even though the melting process reduces molecular weight, thermoplastics retain their original mechanical performance once cooled [23], [24], chiefly if a negligible void content and strong interlaminar bonding are attained by optimizing the in-situ consolidation process [25].

Mechanical fastening and adhesive bonding are conventional methods for joining and repairing metallic components and/or composite materials [26], [27], [28], [29], [30], [31]. However, there are disadvantages associated with mechanical fastening, such as high induced stress concentrations, galvanic corrosion, delamination from rivet drilling, fiber damage in the composites, and weight penalties to the structure due to the addition of metallic fasteners [32], [33], [34], [35], [36], [37]. Adhesive systems require a long curing time, extensive surface preparation is necessary, and the quality of adhesive joints is easily affected by environmental conditions, which constitute significant technical and environmental constraints [8], [38], [39], [40].

Compared to mechanical fastening and adhesive bonding, welding is a more suitable joining technology for thermoplastic composites [41]. Among the various welding techniques currently available for joining and repairing polymeric composites in structural applications, resistance welding is considered one of the most efficient and promising, as it offers advantages such as low operating costs, processing simplicity, and applicability to large structures in a semi-continuous process, such as aircraft wings [34], [42], [43]. Furthermore, the manufacturing of main landing gear doors for the Fokker 50 marked one of the earliest aerospace implementations of resistance welding [3]. The success of this project laid the foundation for scaling up the technology to major structural parts. A prime example is the J-nose on the wing leading edges of the Airbus A340-500/600 and A380 [44], [45], [46], [47]; originally built from several aluminum alloy pieces, this component was re-engineered with polyphenylene sulfide (PPS) composites. This redesign displaced thousands of rivets and achieved a weight reduction of nearly 20% [44].

In resistance welding, an electric current is applied to a component termed the resistive element, or heating element (HE), aiming to significantly increase its temperature through Joule heating. During this process, the temperature of the heating element rises considerably at the interface zone, melting the thermoplastic polymer that is in direct contact with it [48], [49], [50]. When the current flows through the HE, the generated heat originates from the energy dissipated in the resistor which, according to Joule's law, is proportional to the resistance, the square of the current, and the elapsed time, Heat (Q) = I^2^Rt [51], [52], [53].

The process involving the welding of polymeric composites occurs when the supplied energy exceeds the thermal losses within the material and, consequently, the temperature of the laminates begins to rise initially in close proximity to the bonding surfaces and later, if the current duration is extended, deeper into the material. However, it is favorable to maintain the temperature within the so-called heat zone, where it will affect the bonding surface as closely as possible, thereby preventing deformations in the laminate fibers. When the temperature at the bond line increases to a certain point, the thermoplastic matrix begins to melt; once this melting point is reached, the current is turned off, and the assembly is allowed to cool while an appropriate pressure is maintained [39], [41], [54], [55], [56].

Joining thermoplastic composites via resistance welding delivers significant benefits, notably accelerated cycle times, stringent command over processing variables, and highly durable joint interfaces [57], [58]. However, it possesses significant disadvantages and challenges that can limit its application.

There is a high risk of electrical current leakage, which is particularly critical when welding composites that already feature matrices reinforced with conductive carbon fibers, thereby requiring the use of additional insulation and rigorous controls [51], [55]. The process also suffers from major thermal control difficulties; non-uniform temperature distribution along the joint interface can lead to overheating and thermal degradation of the polymer at the edges, or insufficient melting at the weld center, resulting in partially fused zones and the formation of voids (porosity) that weaken the bond [51], [59]. Weld quality is extremely sensitive to operating parameters, meaning that inadequate pressures or times can cause joint deconsolidation, excessive resin extrusion out of the weld area, or even the displacement of the reinforcing fibers [60]. Finally, from a manufacturing and design standpoint, resistance welding requires access to both sides of the surface to apply perpendicular pressure, which precludes its use in confined or restricted spaces, and demands the development of highly specific tooling for each component, complicating tolerance management in large-scale production lines [51].

The development of the proposed equipment is justified by the delivery of a low-cost, autonomous laboratory solution that directly mitigates these limitations through design innovations and closed-loop control. To address the high risk of current leakage, which is particularly critical when welding composites reinforced with conductive carbon fibers, the system strategically adopts a wooden support base that provides structural support and acts as an electrical insulator, preventing current loss through the machine structure. During welding operation, external ceramic insulator plates (not permanent components of the equipment itself) are additionally positioned in direct contact with the composite sample and the welding interface, where they constitute the thermal barrier at the joint, concentrating energy within the joint zone and shielding the wooden base from direct exposure to the elevated interface temperatures. The wooden base therefore acts as a secondary thermal barrier, located further from the heat source. This dual insulation strategy, combined with the precise decimal adjustment of voltage (up to 60 V) and current (up to 24A) provided by the Juntek DPM8624 buck-boost converter coupled with the Huawei R4850G2 power supply, alleviates thermal control difficulties and non-uniform temperature distribution; this prevents both overheating and polymer degradation at the edges as well as insufficient melting at the weld center, thereby reducing the formation of voids and porosity. Furthermore, the extreme sensitivity of weld quality to operating parameters − which frequently causes deconsolidation, excessive resin extrusion, or fiber misalignment − is overcome by integrating an actuator managed by an Arduino microcontroller that processes a load cell signal in real time. This dynamic control of the consolidation force eliminates reliance on external pressure systems and ensures the rigorous and reproducible application of the perpendicular force required on both sides of the surface. Finally, the main structure constructed from aluminum profiles provides lightness and portability to the assembly, transforming a process traditionally dependent on rigid tooling and industrial presses into a flexible and adaptable benchtop system, which facilitates tolerance management and enables the study of dedicated tooling for different component geometries.

## 2 Hardware description

Unlike standard industrial welding stations that rely on closed-source, high-cost proprietary systems, this hardware features a highly customized, open-source architecture tailored for the resistance welding of thermoplastic composites. The structural design replaces heavy, expensive industrial housing with a modular aluminum frame combined with an electrically insulating wooden welding base, specifically engineered to prevent current leakage through the structure during operation. During welding, external ceramic insulator plates (not part of the equipment's bill of materials) are additionally positioned at the sample–electrode contact zone, where they act as the thermal barrier at the joint and shield the wooden structure from the high localized temperatures generated at the weld interface. The core differentiation lies in its cost-effective, automated pressure management system. Instead of relying on manual pneumatic presses or expensive hydraulic systems, this hardware integrates an autonomous feedback loop driven by a low-cost Arduino microcontroller, a linear actuator, and a load cell. This custom integration ensures precise, reproducible pressure control at a fraction of the cost of commercial alternatives. Furthermore, the power supply subsystem − utilizing a Huawei R4850G2 rectifier and a JUNTEK DPM8624 converter − is fully integrated into a custom Python-based GUI. This enables real-time high-fidelity data acquisition, tracking voltage, current, power, temperature via a Type-K thermocouple, and pressure, while providing automated reporting without requiring expensive, specialized laboratory DAQ boards. This integrated platform offers significant advantages in terms of cost efficiency, ease of use, and adaptability compared to pre-existing methods. By replacing five-figure industrial equipment with accessible, off-the-shelf components and open-source hardware, it drastically reduces the financial barrier to advanced composite welding research. Accessibility is further enhanced by the dedicated Python GUI, which streamlines operation, allows researchers to monitor real-time curves such as Voltage vs. Time, and automatically exports standardized PDF reports and synchronized raw data logs, minimizing human error. Additionally, because the platform is fully open-source and modular, it provides an adaptable baseline for future designs. Furthermore, the custom Arduino-Python architecture offers a flexible framework that researchers can modify to implement different automated feedback loops or control algorithms for entirely varied experimental setups.

Beyond the specific application demonstrated in this work, the open and modular nature of the platform makes it useful for a range of standard and novel laboratory tasks, both within and outside the thermoplastic composite’s community:

• **Development of welding windows for new material systems.** Research groups working with matrices, fiber architectures or heating-element configurations that have not yet been characterized can use the equipment to map the interaction between current, consolidation pressure and weld time, correlating the automatically logged power density and resistance with joint performance without requiring dedicated data acquisition hardware;
• **A general-purpose platform for processes combining controlled force with localized Joule heating.** Small-scale consolidation of laminates, heat sealing of polymer films and foils, thermal bonding of textiles and membranes, and thermally activated adhesive curing all rely on the same three capabilities the system already provides − regulated current delivery, closed-loop consolidation pressure and synchronized process logging − and can be addressed by adapting only the sample fixturing;
• **A testbed for process monitoring and control research.** Because the Arduino firmware and the Python interface are fully open, researchers in instrumentation and process control can implement and benchmark alternative strategies on real hardware, such as closed-loop temperature control, resistance-based endpoint detection, or data-driven models trained on the logged process signals;
• **In-situ characterization of resistive heating elements.** Operating at constant current, the continuously recorded voltage is a direct signature of the resistance change of the heating element, so the platform can be used on its own to study the electrothermal behavior of metallic meshes, conductive coatings and nanocomposite heating elements, independently of any welding application;
• **Teaching and capacity building.** The low cost and the complete documentation make it feasible for teaching laboratories and for institutions without access to industrial welding stations to prepare standard-compliant specimens (ASTM D5868) and to expose students to a complete sensor-to-actuator control chain, from load-cell acquisition to automated report generation.

## 3 Design files summary

**Table:** 

**Design file name**	**File type**	**Open source license**	**Location of the file**
Electric	Pdf, tif	CERN-OHL-S v2.0	https://www.https://doi.org/10.17632/8tb37yjp9m.3
Structure part	Dwg, idw, ipt, iam, pdf	CERN-OHL-S v2.0	https://www.https://doi.org/10.17632/8tb37yjp9m.3
Supplementary files	Jpeg, txt, pdf	CC BY 4.0	https://www.https://doi.org/10.17632/8tb37yjp9m.3
Software	Spec, tif, pdf, txt, png, md	MIT License	https://github.com/jonasfrankreis/Open-source-Modular-Resistance-Welding-Equipment-for-Thermoplastic-Composites

The electric design file compiles the complete electrical architecture and physical hardware layout of the equipment, integrating the power supply modules, microcontroller circuitry, and sensor interfaces. It details the system's functional block diagram and the precise wiring schematics required for automated data acquisition and power control, including a simplified electrical block diagram and a simplified electrical schematic that present the voltage levels at each stage of the power chain (127–128 V AC mains input, ≈53.5 V DC rectified bus, and 0–60 V / 0–24 A regulated welding output), the protective-earth grounding topology, and the galvanic separation between the high-power welding circuit and the low-voltage control electronics. The structure part design folder contains the complete mechanical and structural architecture of the equipment, including technical drawings and 3D modeling files of the aluminum frame and insulation base. It provides both ready-to-view PDF documentation and editable CAD files necessary to replicate or modify the physical structure of the welding system. The Supplementary files dataset folder contains auxiliary documentation supporting the equipment validation, including detailed Bills of Materials (BOM) for cost tracking and high-resolution figures of the system. It also provides experimental data logging samples in both raw text format (.txt) and compiled visual summaries (.pdf) to demonstrate the real-time processing of welding parameters. The software section contains the complete source code hosted on the project's GitHub repository, including the Python-based graphical user interface (GUI) and the Arduino firmware configuration. It provides the essential programmatic framework required for automated parameter monitoring, real-time data acquisition, and active feedback control during the welding process.

## 4 Bill of materials summary

The costs listed in bill of materials summary correspond to the prices effectively paid during construction of the prototype described here, in 2026, converted from Brazilian reais at an exchange rate of USD 1.00 = BRL 5.20, and should therefore be regarded as indicative rather than absolute. Prices of identical or functionally equivalent components vary substantially between regions as a result of local taxation, import duties, shipping costs and exchange-rate fluctuation, and the supplier links provided identify the specific units purchased rather than exclusive or recommended sources.

Of the 37 items listed, 20 do not depend on any particular supplier: 7 are machined from the technical drawings provided in the design file repository, 4 were produced from reclaimed material, and 9 are generic hardware items − fasteners, brackets and levelling feet − obtainable from any local retailer. Most of the remaining items are widely distributed commodity electronics, including the microcontroller board, the load-cell amplifier, the H-bridge driver, the load cell and the display, for which functionally equivalent substitutes are available worldwide from multiple suppliers.

Only the programmable DC power supply (AE) and the rectifier module (AG) correspond to specific commercial models; both are internationally distributed, and in principle any regulated DC source capable of delivering up to 60 V and 24 A under serial or radio-frequency communication may be substituted, provided the communication protocol implemented in the control software is adapted accordingly. Users replicating the equipment in other regions should therefore expect the total cost to differ from the value reported here, while the reproducibility of the design itself, which rests on the supplied CAD, electrical and software files, remains unaffected.

## 5 Build instructions

### 5.1 Structural Part

**Fig. 1:**
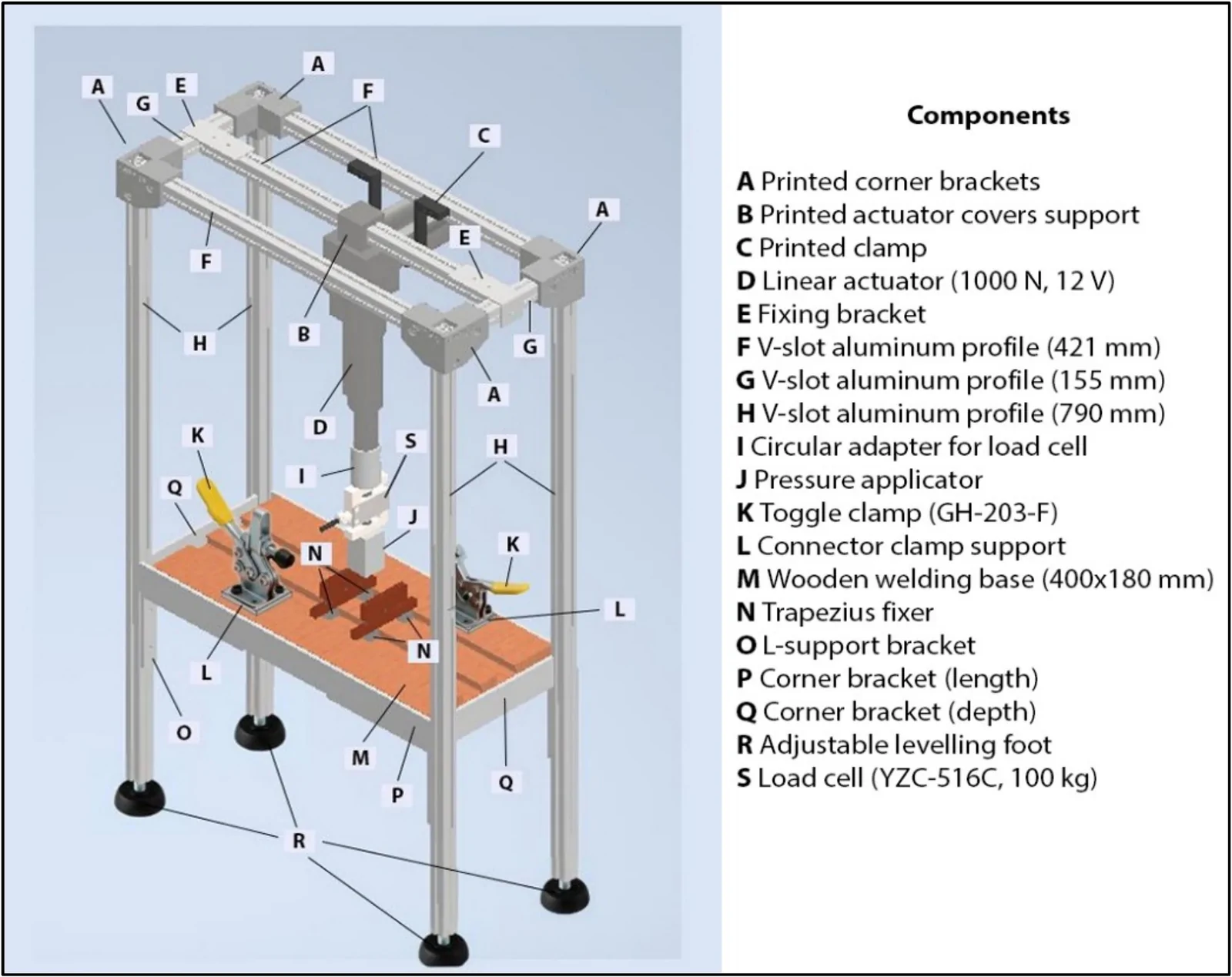
Main structural frame of the resistance welding machine, assembled from aluminum profiles (F, G, H) and printed corner brackets (A), designed to support the pressure application system, electrode base, and electrical enclosures in a modular configuration.

**Fig. 2:**
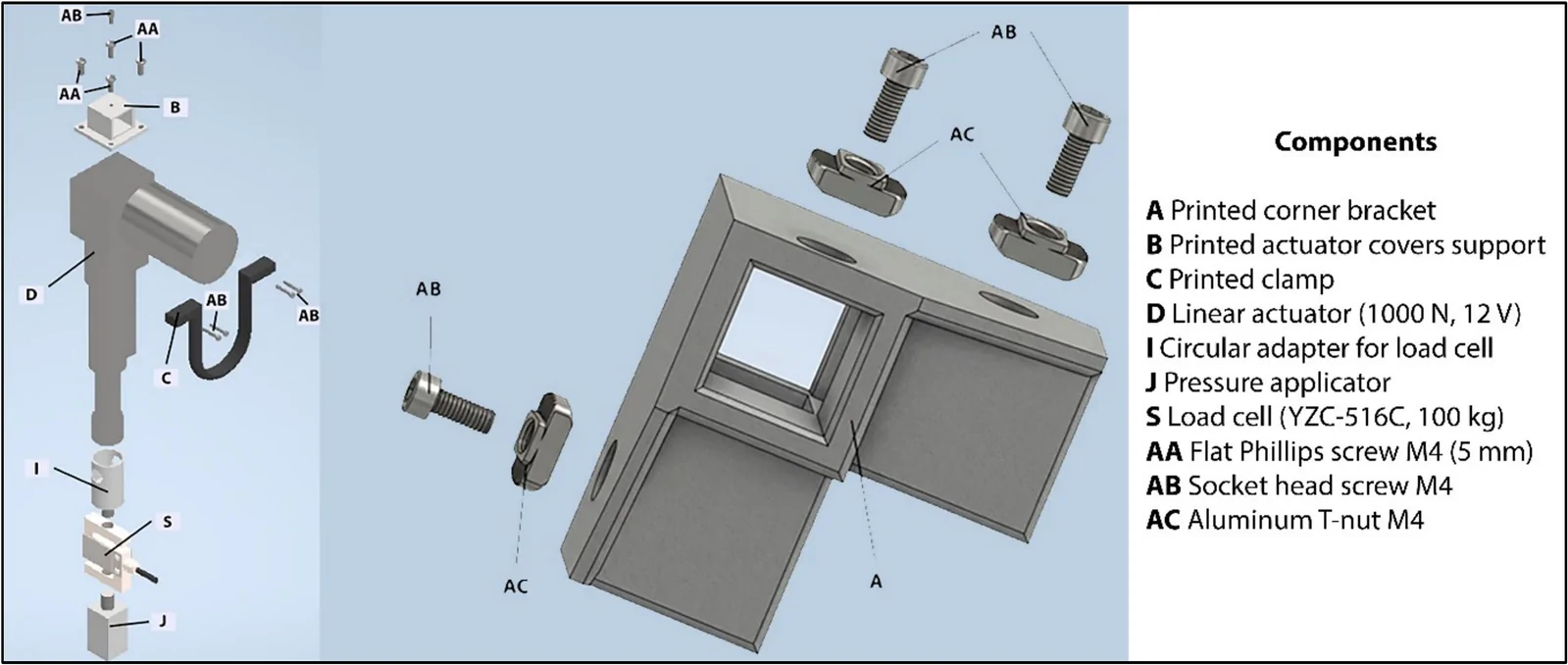
Pressure application system comprising the electric linear actuator (D), load cell (S), and custom pressure applicator (J), mounted centrally on the frame via printed supports (A, C) and L-brackets (O) for stable load transmission.

**Fig. 3:**
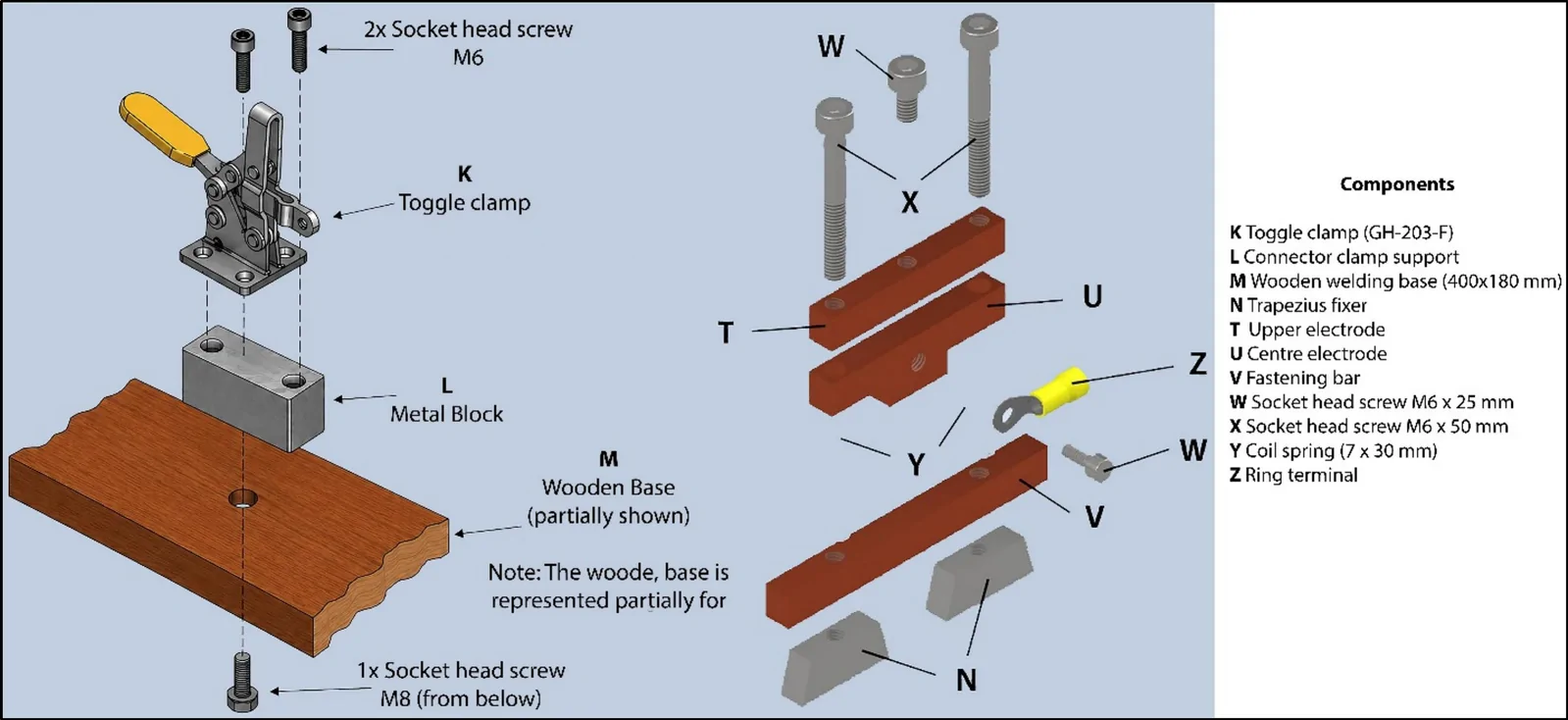
Sample base and electrode assembly, including the welding base (M) with trapezius fixers (N), dual electrode pairs (T, U) with spring-loaded adjustment (Y), fastening bars (V), and toggle clamps (K) with interface blocks (L) for specimen securing.

**Fig. 4:**
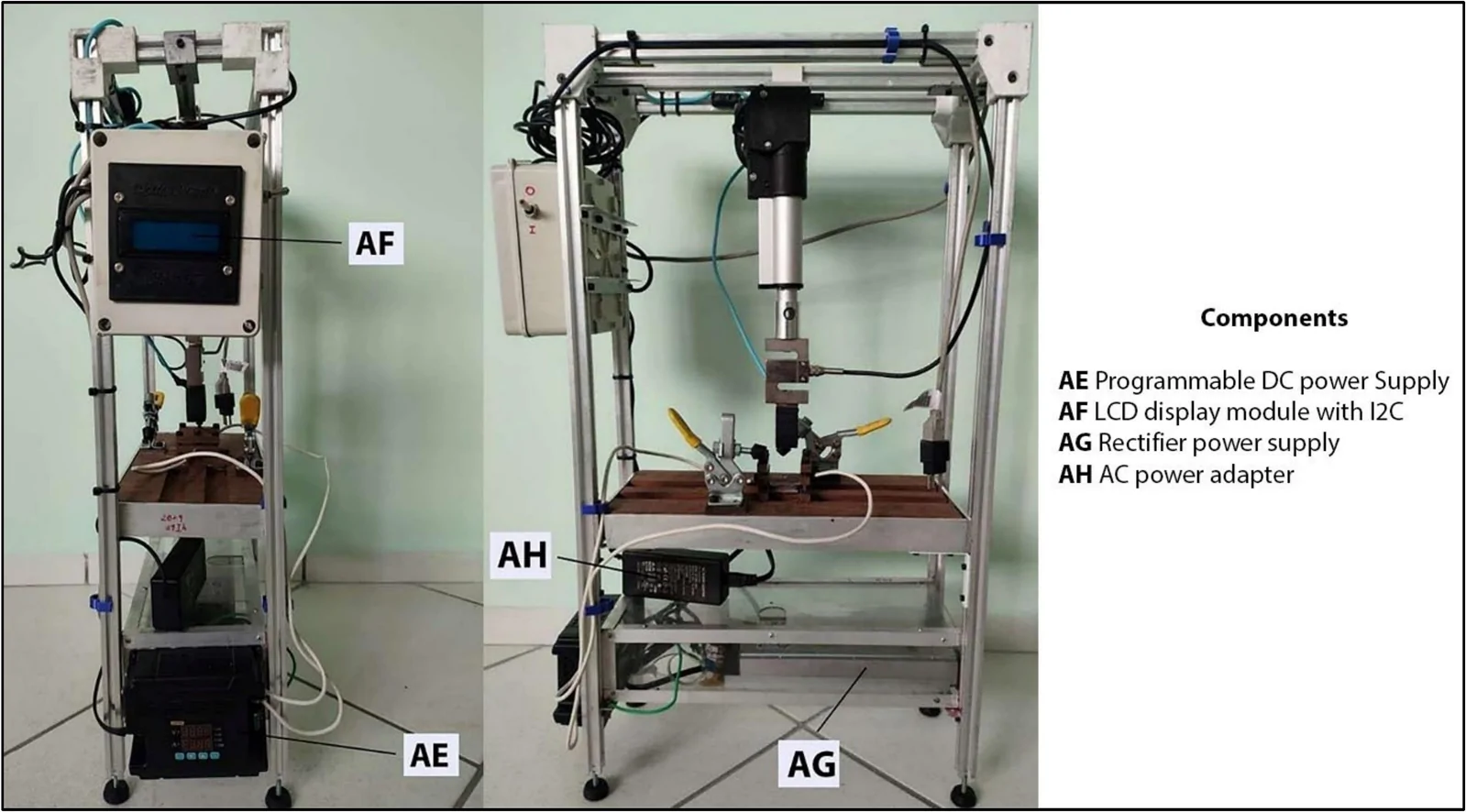
Integration of electrical enclosures on the machine frame, showing mounting positions between profile elements and control modules.

**Fig. 5:**
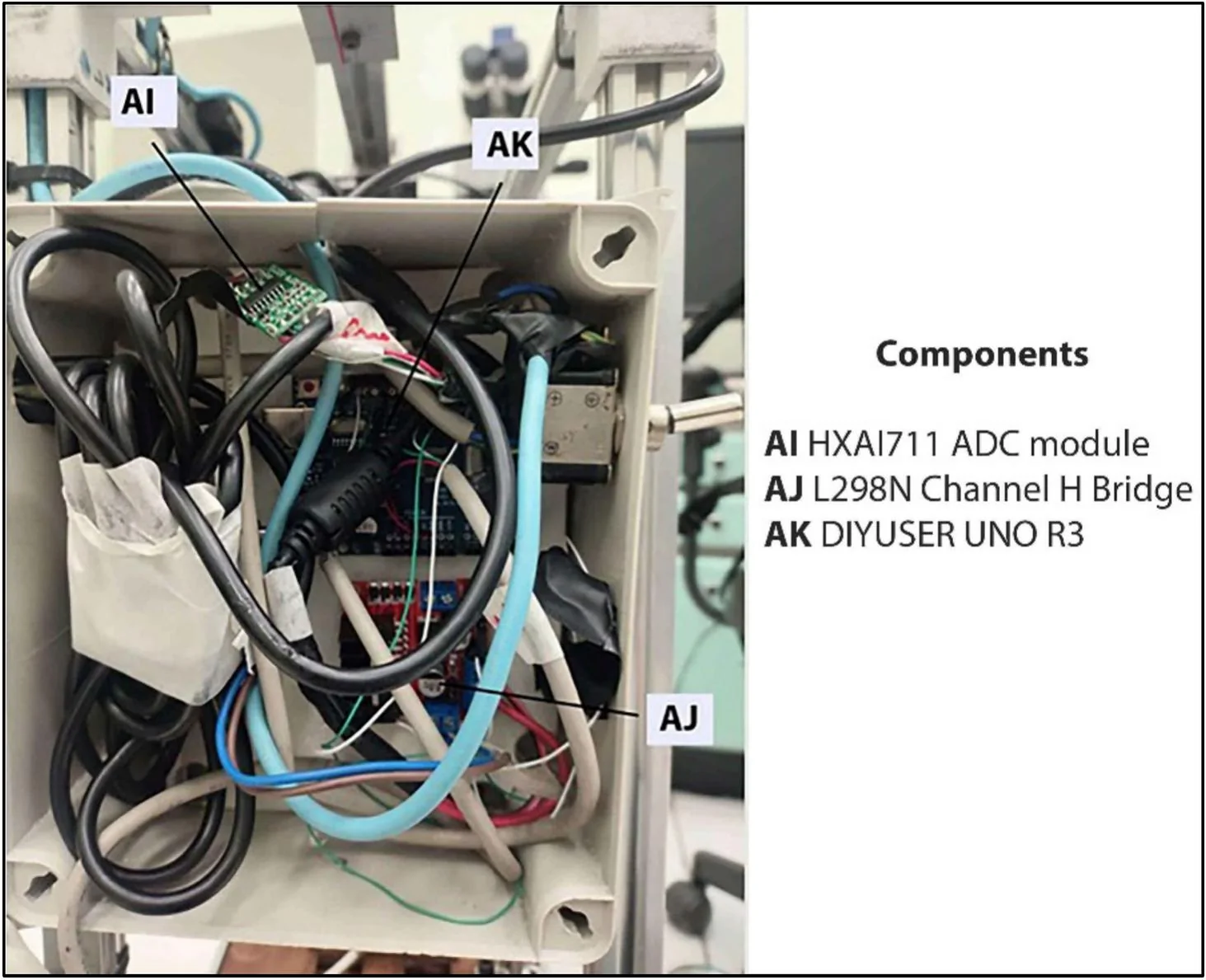
Data acquisition and electrical command box coupled to the structural frame.

**Fig. 6:**
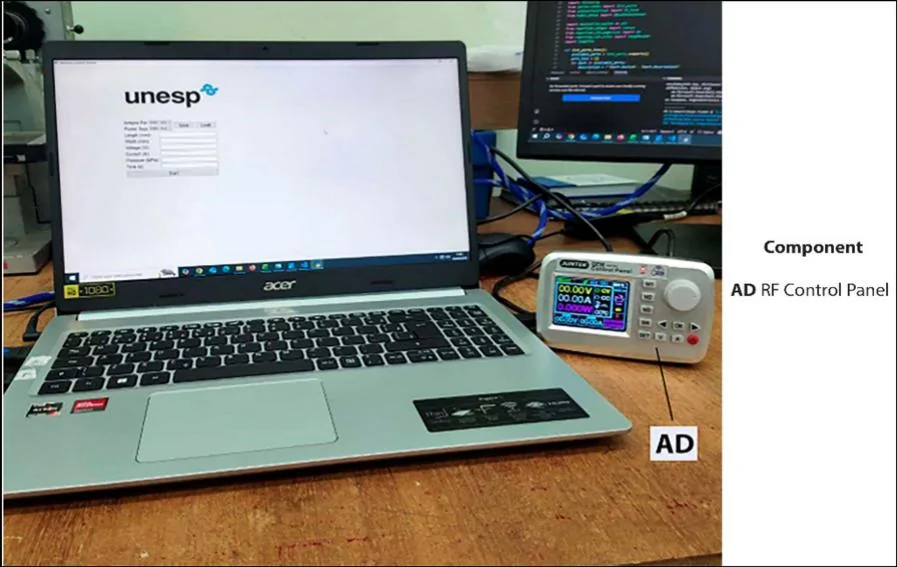
RF control panel and programmable DC power supply mounted on the frame side, enabling wireless current regulation, monitoring, and communication with the data acquisition system.

The structural part of the welding machine (Fig. 1) was designed for the assembly of components such as the pressure application system (Fig. 2), sample bases, and electrodes (Fig. 3), resulting in a modular design that allows for easy adaptation as needed. The frame also accommodates the coupling of electrical enclosures (Fig. 4), data acquisition boxes (Fig. 5), and RF Control Panel (Fig. 6). Detailed descriptions for each component can be found in Table 1.

**Table 1:** Structural components of the modular welding machine.

***Designator***	***Component***	***Number***	***Cost per unit − USD***	***Total cost −*** ***USD***	***Source of materials***	***Material type***
A	Printed Corner Brackets (103 g)	4	$2.04	$8.17	https://3dfila.com.br/?s=PETG&post_type=product	PETG
B	Printed actuator covers support (35 g)	1	$0.69	$0.69	https://3dfila.com.br/?s=PETG&post_type=product	PETG
C	Printed Clamp (77 g)	1	$1.53	$1.53	https://3dfila.com.br/?s=PETG&post_type=product	PETG
D	1000 N DC 12 V Linear Actuator Push 100Kg	1	$115.8	$115.8	https://www.mercadolivre.com.br/atuador-linear-pistao-eletrico-100mm-10cm-12v-ate-100kg/up/MLBU3919797622#polycard_client=search-desktop&be_origin=backend&search_layout=grid&position=3&type=product&tracking_id=f263a36f-ed00-44f7-b23d-f7126e259452&wid=MLB6647387384&sid=‘search	Steel
E	Fixing bracket	2	−	−	Waste	Steel
F	V-Slot Aluminum Profile Extrusion (421 mm)	3	$5.98	$17.94	https://lista.mercadolivre.com.br/perfil-de-aluminio-estrutural-v-slot-20x40-tipo-openbuilds#redirectedFromVip	Aluminum
G	V-Slot Aluminum Profile Extrusion (155 mm)	2	$2.20	$4.40	https://lista.mercadolivre.com.br/perfil-de-aluminio-estrutural-v-slot-20x40-tipo-openbuilds#redirectedFromVip	Aluminum
H	V-Slot Aluminum Profile Extrusion (790 mm)	4	$11.22	$44.8	https://lista.mercadolivre.com.br/perfil-de-aluminio-estrutural-v-slot-20x40-tipo-openbuilds#redirectedFromVip	Aluminum
I	Circular adapter for load cell connection	1	−	−	Machined Part	Stainless steel
J	Pressure applicator	1	−	−	Machined Part	Stainless steel
K	GH-203-F Hand Tool Toggle Clamp Upgrade Horizontal	2	$4.10	$8.20	https://pt.aliexpress.com/item/1005005252283243.html?gatewayAdapt=glo2bra	Steel
L	Connector clamp support	2	−	−	Machined Part	Steel
M	Resistance welding wood base (400x180mm)	1	−	−	Waste	Wood
N	Trapezius fixer	4	−	−	Machined Part	Steel
O	L-Support Bracket	4	$0.80	$3.20	Local store	Stainless steel
P	Corner bracket length	2	−	−	Waste	Steel
Q	Corner bracket depth	2	−	−	Waste	Steel
R	Adjustable Lathe Leg	4	$0.81	$3.24	https://www.mercadolivre.com.br/06-sapata-pe-nivelador-rosca-m6-pezinho-regulavel-movel-mesa/up/MLBU1753129605?pdp_filters=item_id%3AMLB5065575314&matt_tool=38524122#origin=share&sid=share&wid=MLB5065575314	Steel
S	YZC-516C Tension and Compression Load Cell 100Kg	1	$21.00	$21.00	https://www.lollette.com/yzc-516c.html	Steel
T	Upper electrode	2	−	−	Machined Part	Copper
U	Center electrode	2	−	−	Machined Part	Copper
V	Fastening bar	2	−	−	Machined Part	Copper
W	Hexagon Allen Hex Socket Cup Head Screw Bolts M6 25 mm	4	$0.13	$0.52	Local store	Steel
X	Hexagon Allen Hex Socket Cup Head Screw Bolts M6 50 mm	10	$0.13	$1.30	Local store	Steel
Y	Coil Spring 7x30mm	4	$0.75	$3.00	Local store	Steel
Z	Ring Terminal	2	$0.02	$0.04	Local store	Steel
AA	Stainless Steel Flat Phillips Screw (5 mm)	4	$1.46	$5.84	Local store	Stainless steel
AB	Hexagon Allen Hex Socket Cup Head Screw Bolts M4	53	$0.10	$5.30	Local store	Steel
AC	Series Industrial Aluminum T Nuts M4	53	$0.2	$10.60	Local store	Aluminum
AD	RF Control Panel	1	$24.75	$24.75	https://alishort.com/tpP81	Electronic
AE	JUNTEK DPM8624-485RF 60 V 24A Programmable DC Power Supply	1	$75.84	$75.84	https://alishort.com/tpP81	Electronic
AF	LCD Display Module with IIC/I2C Serial Interface Adapter	1	$6.33	$6.33	https://shorturl.at/tlurN	Electronic
AG	R4850G2 Communication Power Supply	1	$112.91	$112.91	https://shorturl.at/lMlJN	Electronic
AH	AC Power Adpater ADP-90FB	1	−	−	Local store	Electronic
AI	HX711 24-bit ADC Module On-board TL431	1	$2.02	$2.02	https://shorturl.at/o0Iw5	Electronic
AJ	L298N Dual Channel H Bridge	1	$0.95	$0.95	https://shorturl.at/lP7AH	Electronic
*AK*	DIYUSER UNO R3 ATmega328P Board with USB	1	$2.83	$2.83	https://shorturl.at/ezXAP	Electronic

### 5.2 Step-by-step assembly instructions

• Main Frame Assembly: Assemble the aluminum profile frame by securing the two F components, the four H components, and the two G components. To do this, use four Printed Corner Brackets (A) and establish the mechanical fastening using M4 screws (AB) and M4 nuts (AC).
• Actuator Support Installation: The Printed actuator covers support (B) must be inserted into the final F element prior to its definitive fastening. Align it to the center of the F element's length and secure it using one M4 screw (AB) at its center and four Flat Phillips Screws (AA) at the outer edges.
• Base Leveling: Thread the four Adjustable Lathe Legs (R) (one into each H element) to allow for the proper leveling of the frame.
• Central Element Fastening: Secure the final F element to the center of the G elements' length, using the Fixing bracket (E), M4 screws (AB), and M4 nuts (AC).
• Load System Stabilization: The load application elements must be secured to the frame via the Printed Corner Brackets (A) and support C, using four M4 screws (AB) to ensure the stability and proper alignment of the assembly.
• Support Elements Assembly and Height Adjustment: Fasten the Q elements to the H elements using two M4 screws (AB) with M4 nuts (AC) for each Q element. The mounting height must be sufficient to allow for sample handling beneath element J, while respecting the maximum stroke of the actuator (D). Next, couple the P elements to the Q elements (one on each side) and secure them by applying the L-support (O) between element P and element H, using four M4 screws (AB) and four M4 nuts (AC) for each L-support. Ensure perfect alignment of all parts described in this step.

### 5.3 Pressure application system

The assembly of the load application system is centralized and relatively straightforward, facilitating the replacement of its components should the user need to increase the applied pressure area. The use of the L-support (O) is critical to prevent bending effects during load application, as it stabilizes the frame and assists in transmitting the reaction force directly to the machine chassis. Although the actuator can be replaced by a NEMA stepper motor-based system, an electric actuator will be utilized in this specific configuration.

• Load Cell Fastening: The load cell (S) must be secured to the electric actuator (D), which should already be pre-assembled onto supports A and C. If necessary, use a threaded adapter to enable the coupling of the load cell to the electric actuator, following the same method applied to element I in this assembly.
• End Actuator Installation: At the free end of the load cell, attach the pressure applicator (J). This component can be machined or custom-manufactured according to the surface area requirements specified for joining the parts.

### 5.4 Base and electrode assembly

• Fixer Positioning: The four Trapezius fixers (N) must be fitted into the slots of the welding wood base (M), positioning two on one side (in separate slots) and the other two on the opposite side (also in separate slots).
• Electrode Assembly Pre-mounting: Position parts T and U of the electrode and insert the two M6 screws (X) to keep the parts aligned. Next, fit the springs (Y) onto the shanks of the X screws. This entire subassembly (T, U, X, and Y) must be placed over the Fastening bar (V), advancing the fastening until the screw shanks completely pass through the system.
• Electrode Fastening and Adjustment: Position the mounted assembly over the two corresponding Trapezius fixers (N), which run parallel to each other, and secure them by applying the appropriate torque to the X screws. Screw W must be inserted and secured into the center hole of the Upper electrode (T), but final tightening should only be performed when the resistive element is properly positioned and ready for the start of welding operations.
• Symmetry and Modulation: Repeat the exact same procedure for the second electrode located on the opposite side. It is worth noting that the distance between the electrodes is variable and can be easily adjusted according to the geometry or the type of specimen to be welded.

### 5.5 Toggle clamps installation

• Mechanical Clamp Assembly: The GH-203-F Hand Tool Toggle Clamp Upgrade Horizontal (K) units must be installed onto the base. For this design, a block interface (L) positioned between the base (M) and the clamp (K) was adopted.
• Fastening and Angular Adjustment: Secure the clamp (K) to block L. Subsequently, fasten block L to the base (M) using an M8 screw inserted from the bottom of the base until it engages the threads of the block. This configuration allows the clamp (K) to be rotated and locked at different positions and angles as required for clamping. Repeat the same procedure on the opposite side to install the second clamp.
• Base Fitting: The assembled base must be inserted into the main frame through the longitudinal clearance delimited by the opposing structural elements H. Ensure it is positioned firmly resting on the base support structural elements (P and Q).

### 5.6 Electrical system coupling

The project is designed to operate independently of the data acquisition system, electrical commands, and power supply, providing greater versatility to the user. However, if a system integrated with the equipment is preferred, the data acquisition system can be mounted inside an electrical enclosure and coupled to the frame via the H elements, using M4 screws and nuts (AB and AC). The system's power supply can be housed in an acrylic enclosure attached to aluminum corner brackets and secured to the frame by fastening it with M4 screws and nuts (AB and AC) onto the structural H elements. The advantage of utilizing this arrangement is that it increases structural stability through mechanical interlocking. If the user opts for this mounting configuration, it is recommended to install it below the base assembly comprised of elements M, O, P, and Q, near the end of the frame.

Electrical enclosures were adopted in this specific project and coupled to the frame. Three elements related to the electrical and data acquisition section were used the LCD Display Module with IIC/I2C Serial Interface Adapter (AF) and the JUNTEK DPM8624-485RF 60 V 24A Programmable DC Power Supply (AE), both secured by M4 screws and nuts (AB and AC) to the side of the frame between the H elements; and the R4850G2 Communication Power Supply (AG), secured to the four structural H elements with M4 screws and nuts (AB and AC).

This unit is located below the base assembly comprised of elements M, O, P, and Q, housing an electrical power supply (AG) with a dual-voltage input and a DC 60 V 24A output. The power supply input cable originates from an external AC power outlet/source, passes through a toggle switch, and connects to the power supply input terminal. The output is converted to DC and connected via two cables (positive and negative poles) to the *Programmable DC Power Supply* (AE) for electrical power distribution. A grounding cable is connected between the mechanical joints of the frame and the electrical power supply enclosure using a ring terminal positioned between the frame and the M4 fastening screw. This cable connects to the electrical power supply ground, which is tied into the grounding system of the AC power supply source. A grounding electrical connection must also be established using a ring terminal or washer attached to the fastening thread between the pressure applicator block (J) and the load cell (S).

This system is fastened to the lower side of the frame between two H elements using M4 screws and nuts. This system is responsible for reading and controlling the electrical current output, as well as handling wireless communication with the software. It is powered by element AG, and its output is connected via two 6 mm^2^ cables routed to each electrode (N) using a ring terminal (Z) secured with an M6 screw.

### 5.7 Data acquisition and electrical command box

• Arduino General Power Supply (AK)
• USB Connection: Connect the notebook's USB cable to the Arduino Uno's USB port for serial communication and initial development power.
• External Power: Connect the P4 plug of an external power supply/battery directly into the Arduino's power jack input to ensure stable power delivery to the peripherals.

### 5.8 LCD display Module I2C wiring (AF) and HX711 amplifier Module wiring (AI)

The display utilizes I2C communication, requiring only 4 wires connected to the Arduino:

• GND (Black): Connect to the Arduino GND pin (upper header, adjacent to pin 13).
• VCC (Red): Connect to the Arduino 5 V pin (lower power header).
• SDA (Green): Connect to digital pin A4 (or to the dedicated SDA pin on the upper left edge).
• SCL (Gray): Connect to digital pin A5 (or to the dedicated SCL pin on the upper left edge).
• GND (Black): Connect to the Arduino GND pin (lower header).
• DT / Data (Dark Green): Connect to the Arduino digital pin 3.
• SCK / Clock (Dark Gray): Connect to the Arduino digital pin 2.
• VCC (Red): Connect to the Arduino 5 V pin (sharing the power rail).

Connect the 4 colored wires exiting the load cell directly to the corresponding terminals of the HX711:

• E+ →Red wire
• E- →Black wire
• A-→ White wire
• A+ →Green wire

### 5.9 Motor driver wiring (AJ)

• Control Pin 1 (Light Green): Connect from the Arduino digital pin 5 to the first signal pin of the driver (IN1).
• Control Pin 2 (Light Gray): Connect from the Arduino digital pin 6 to the second signal pin of the driver (IN2).
• Connect the positive pole (+) of an external power supply/battery to the first terminal of the driver (VMS/12 V).
• Connect the negative pole (−) of the external battery to the central terminal of the driver (GND).

Connect the pair of driver power output wires (front 2-way terminals) directly to the terminals of actuator motor D (Brown and Blue wires).

Attention: The Arduino GND must always be connected to the common GND of the motor driver's low-voltage (12 V) power supply, to provide a stable reference for the digital control signals (IN1, IN2), regardless of the driver's internal regulator state. This connection is limited exclusively to the low-voltage actuator driver circuit (AJ) and must never be extended to the high-power welding bus. The welding circuit (Huawei R4850G2 rectifier and JUNTEK DPM8624 converter, up to 60 V DC / 24 A) is electrically independent from the Arduino and is monitored and controlled exclusively through the wireless RF Control Panel link, so no direct galvanic connection, ground loop, or back-EMF path exists between the low-voltage control electronics and the high-power welding circuit.

## 6 Operation instructions

Operation of this equipment involves electrical, mechanical, and thermal hazards. Strict adherence to the following precautions is mandatory to prevent injury, equipment damage, or fire:

• Electrical hazards: The equipment is powered from a 127–128 V AC mains supply (standard Brazilian residential/laboratory circuit), which is internally rectified and regulated to a DC welding output of up to 60 V and 24 A delivered to the electrodes. Always verify proper grounding of the equipment and power supply. Never connect or disconnect cables with the power source energized. Inspect all cables and connectors for damage before use. Work in dry conditions only.
• Mechanical hazards: The actuator applies high force (up to 1.5 MPa). Keep hands, fingers, and any body parts clear of the actuator path and clamping area during operation. Use the provided “connector clamp support” correctly to avoid unexpected movement.
• Thermal hazards: Resistive heating during welding can produce localized high temperatures. Allow the sample and electrodes to cool sufficiently before handling. Use heat-resistant gloves when removing samples.
• In case of emergency, immediately stop the process via the software “Stop” command, disconnect the main power supply, and follow laboratory emergency procedures.

### 6.1 Step-by-step operating procedure

**Fig. 7:**
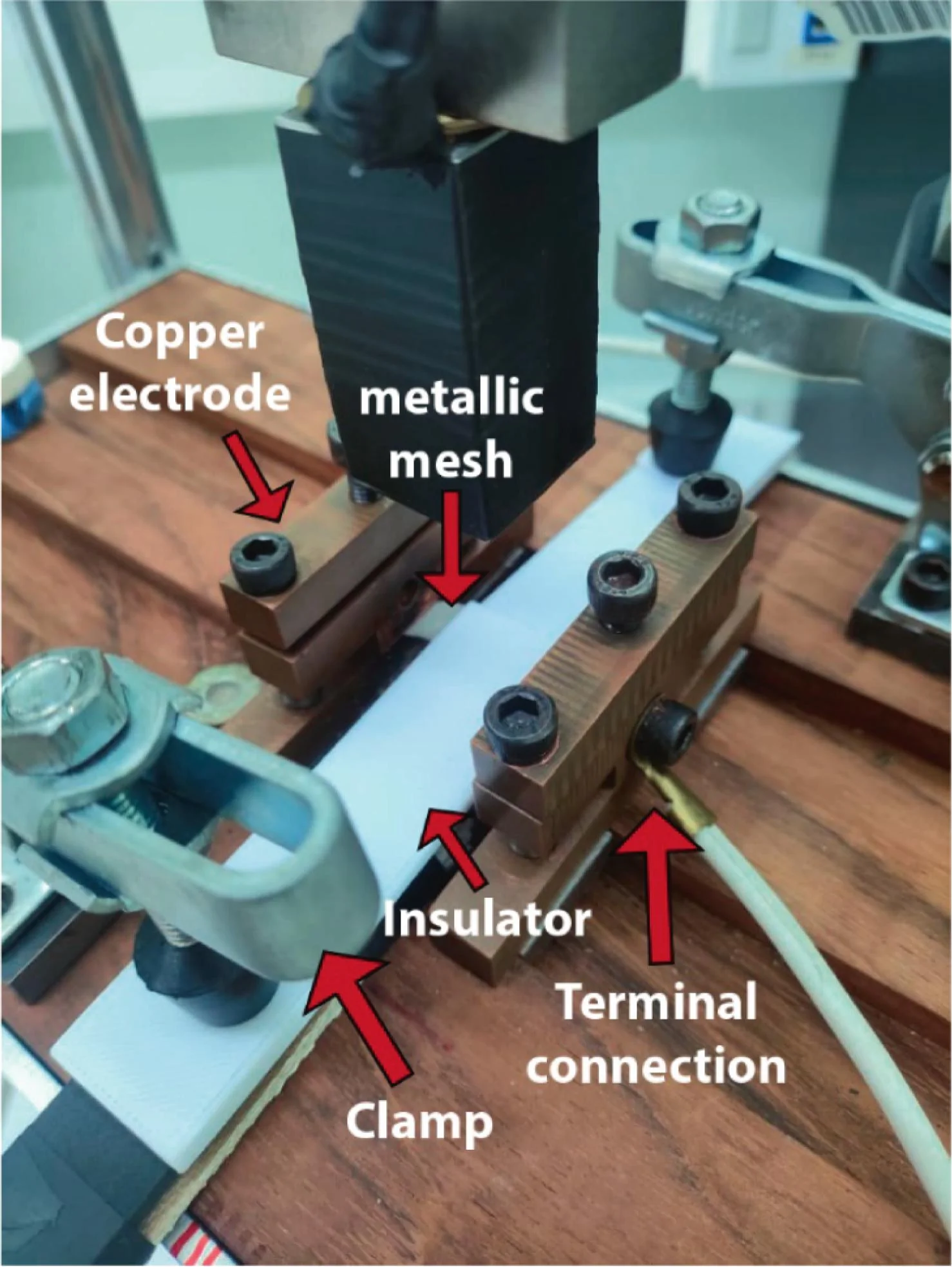
Sample assembly setup.

**Fig. 8:**
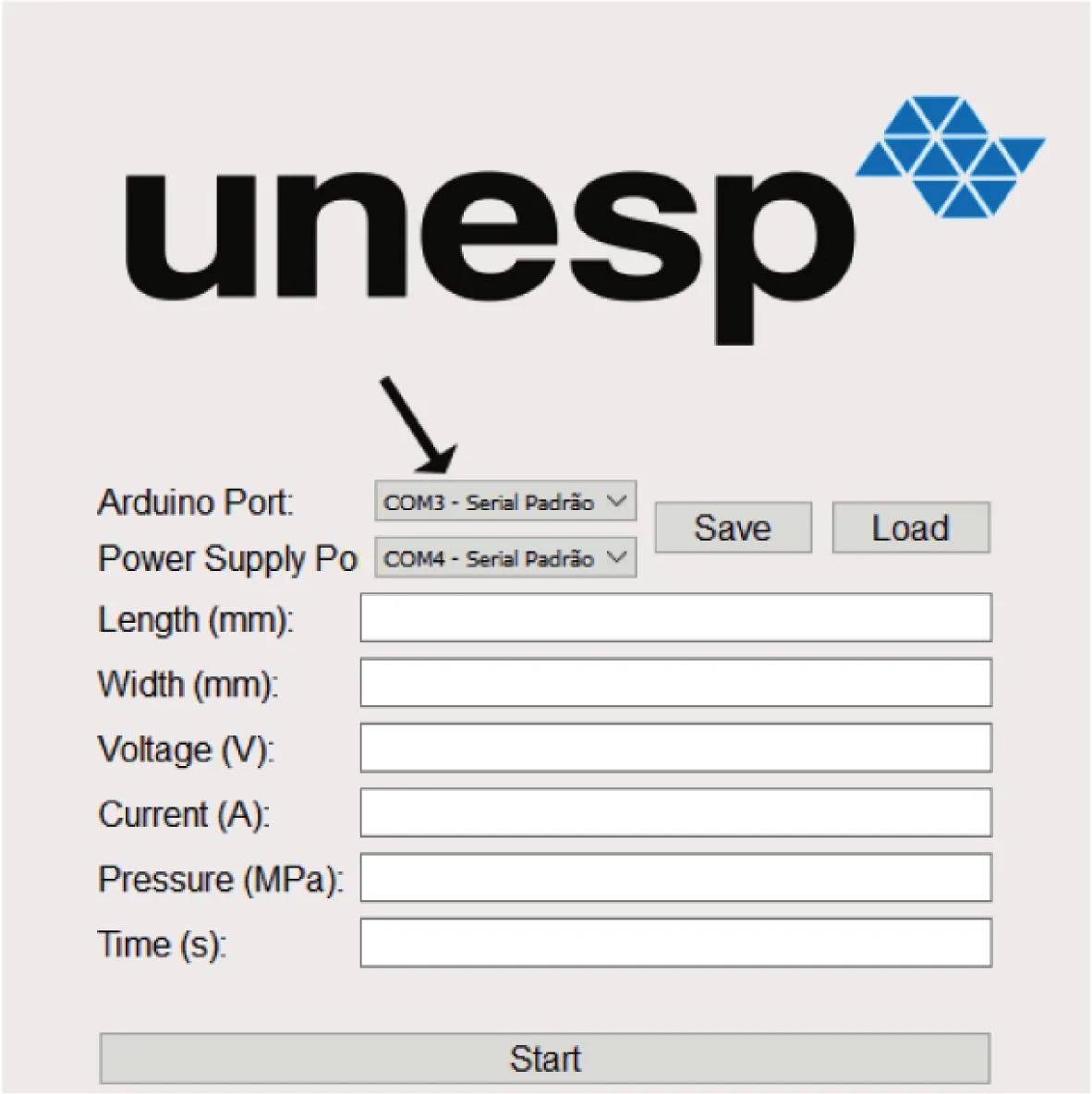
Main software interface showing automatic USB connection status and ready state.

a) Sample Positioning: Connect the R4850G2 power supply to the JUNTEK DPM8624-485RF Programmable DC Power Supply. Connect the positive and negative terminals to the copper electrodes. Position external ceramic insulator plates − not components of the equipment itself − at the wood base and at the welding interface, respectively, to provide additional thermal and electrical protection to the wooden base. Insert the prepared thermoplastic composite sample between the metallic mesh and the copper electrode, resting on the ceramic insulator plates. To prevent displacement or misalignment during pressure application, secure the sample firmly using the “connector clamp support” (Fig. 7).

b) Electrical and Control Connections: Connect the main power cable from the power supply to the equipment power inlet. The equipment's AC power inlet is designed to operate from a 127–128 V AC mains supply; the DC welding output delivered to the electrodes is separately regulated up to 60 V and 24 A by the JUNTEK DPM8624 converter (Section 4). Connect the USB cable from the Arduino board to the computer. Connect the USB cable from the “RF Control Panel” controller to the computer. Warning: Confirm that the power supply is switched OFF before making any electrical connections.

c) Software Launch and Connection Verification: Open the control software. Upon launch, the program automatically detects and establishes communication with the Arduino board and RF Control Panel via USB. Successful connection is indicated on the software interface (Fig. 8).

d) Parameter Configuration: Enter the following parameters in the software interface:

• Sample length and width (mm).
• Voltage (V).
• Current (A) − maximum 24 A with current equipment configuration.
• Pressure (MPa) − maximum 1.5 MPa with current equipment configuration.
• Welding time (duration).

Verify all entered parameters against the experimental protocol. The control software automatically imposes upper safety limits for current (maximum 24 A) and pressure (maximum 1.5 MPa) to protect the equipment and ensure safe operation. Note that the applied voltage is user-defined. However, it varies dynamically according to the set current and the instantaneous electrical resistance of the metal mesh-sample circuit.

e) Process Initiation and Monitoring: Confirm that the sample is correctly positioned and secured, all personnel are clear of the work area, and parameters are correctly entered. Press the “Start” button. The actuator will advance and contact the sample. The system will apply and stabilize the target pressure. Once pressure stabilization is confirmed, current is automatically released through the electrode–mesh assembly, initiating resistive (Joule) heating at the weld interface. Monitor real-time process variables (pressure, current, voltage, and time) on the software interface throughout the cycle. The process terminates automatically after the programmed time or upon reaching the defined endpoint conditions. The current is cut off first, followed by controlled pressure release and actuator retraction.

f) Sample Removal and System Shutdown: Wait for the software to indicate cycle completion and safe status. Carefully remove the connector clamp support and extract the welded sample using heat-resistant tools or gloves. Close the software. Disconnect the USB cables from the computer, followed by the main power cable (after confirming the power supply is off). Clean the electrode and mesh surfaces according to the preparation protocol described in Section 7 before subsequent runs, and return all tools to their designated storage locations.

## 7 Validation and characterization

Voltage measurements of the power supply system were calibrated using a Rigol DM3068 6.5-digit bench multimeter, while current measurements were calibrated with an Itech IT8512 + electronic load. Temperature monitoring inside the composite joint was performed with Type K thermocouples (model TP-01), suitable for the 50–400 °C range, connected to a Fluke True-RMS 117 digital multimeter.

To demonstrate operation of the developed resistance welding hardware and software and to characterize its performance for a relevant scientific application, three replicate lap-joint specimens of glass fiber-reinforced polyetherimide (GF/PEI) thermoplastic composite were welded according to ASTM D5868. The specimens (25.4 × 12.7 mm) were processed using the following parameters: 10 V, 14 A, 1 MPa applied pressure, and 100 s weld time.

**Fig. 9:**
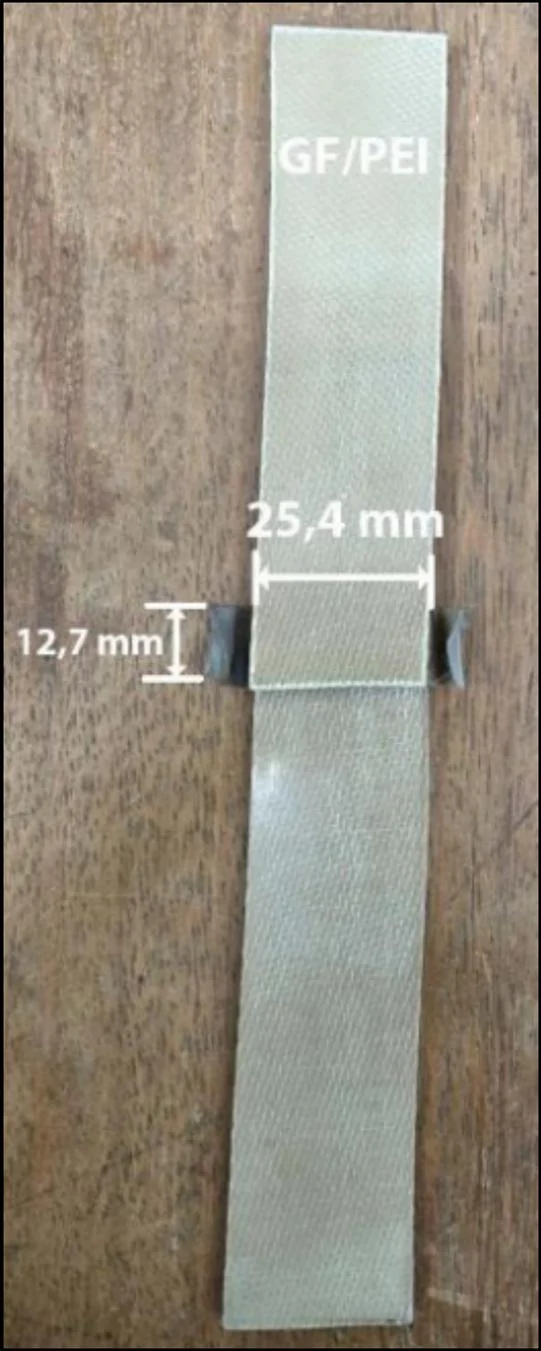
Visual inspection of welded GF/PEI lap-joint specimens showing joint integrity after resistance welding.

The heating element used in the present study was an AISI 304 stainless steel woven wire mesh with a wire diameter of 40 µm and a mesh opening of 89 µm, cut into strips 12.7 mm in width and 80 mm in length, with a free span of 50 mm between the electrodes. Prior to welding, the meshes were cleaned in an ultrasonic bath with ethanol for 15 min to remove oil and grease residues, and the copper electrode contact faces were abraded with P600 abrasive paper and degreased with ethanol before each welding series, in order to remove surface oxide and ensure reproducible contact resistance at the electrode-mesh interface. This heating element configuration was selected on the basis of previous studies in which different metallic mesh configurations were evaluated and compared [46], [61]; it should not be regarded as exclusive, since other combinations of wire diameter and mesh opening may be employed, as may non-metallic heating elements such as carbon fibers [31]. Visual inspection of the welded joints (Fig. 9) confirmed integrity, with no evidence of excessive pressure or current-induced degradation of the joint.

**Fig. 10:**
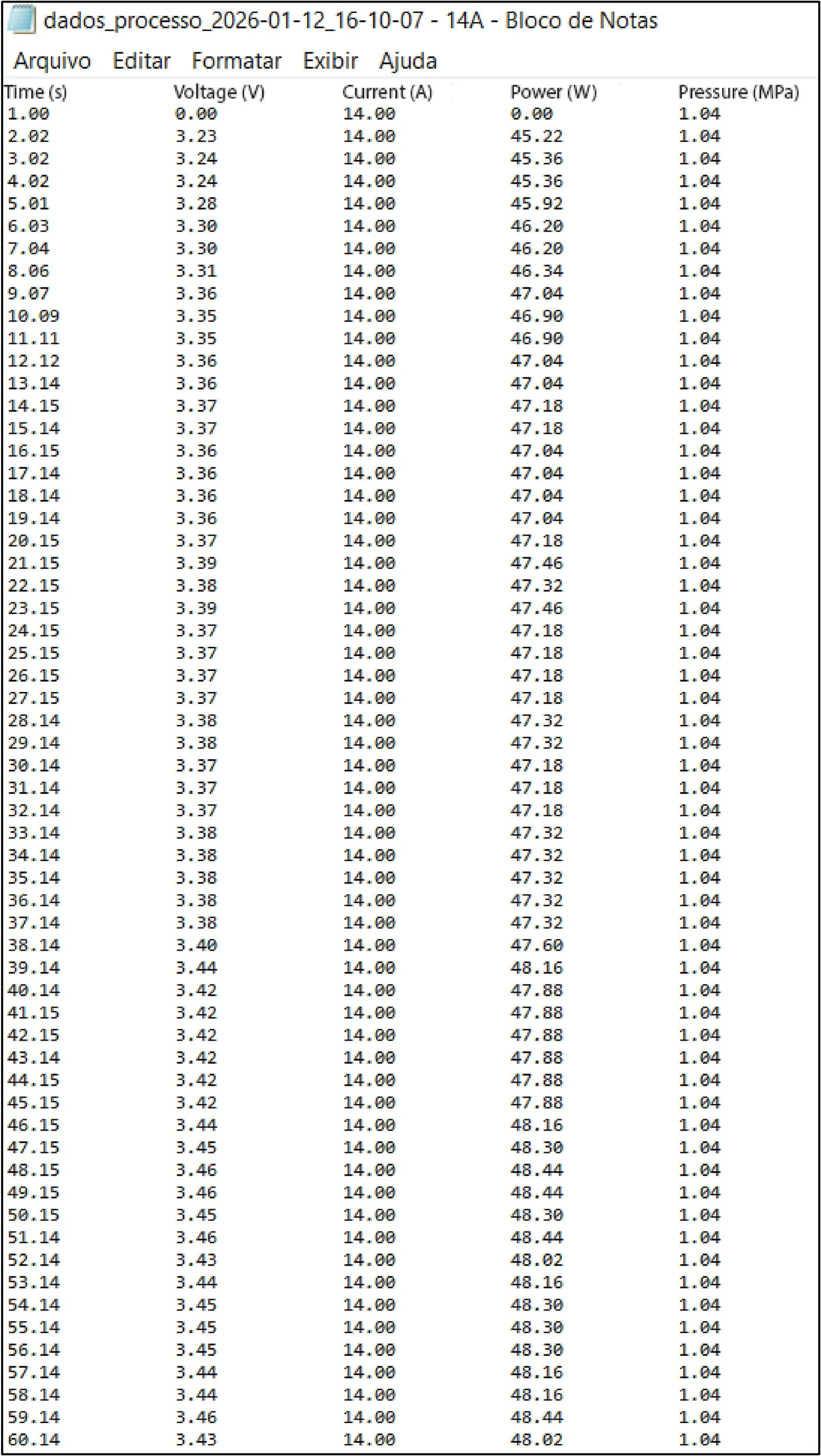
Example of real-time process data logged by the software and exported in.txt format.

**Fig. 11:**
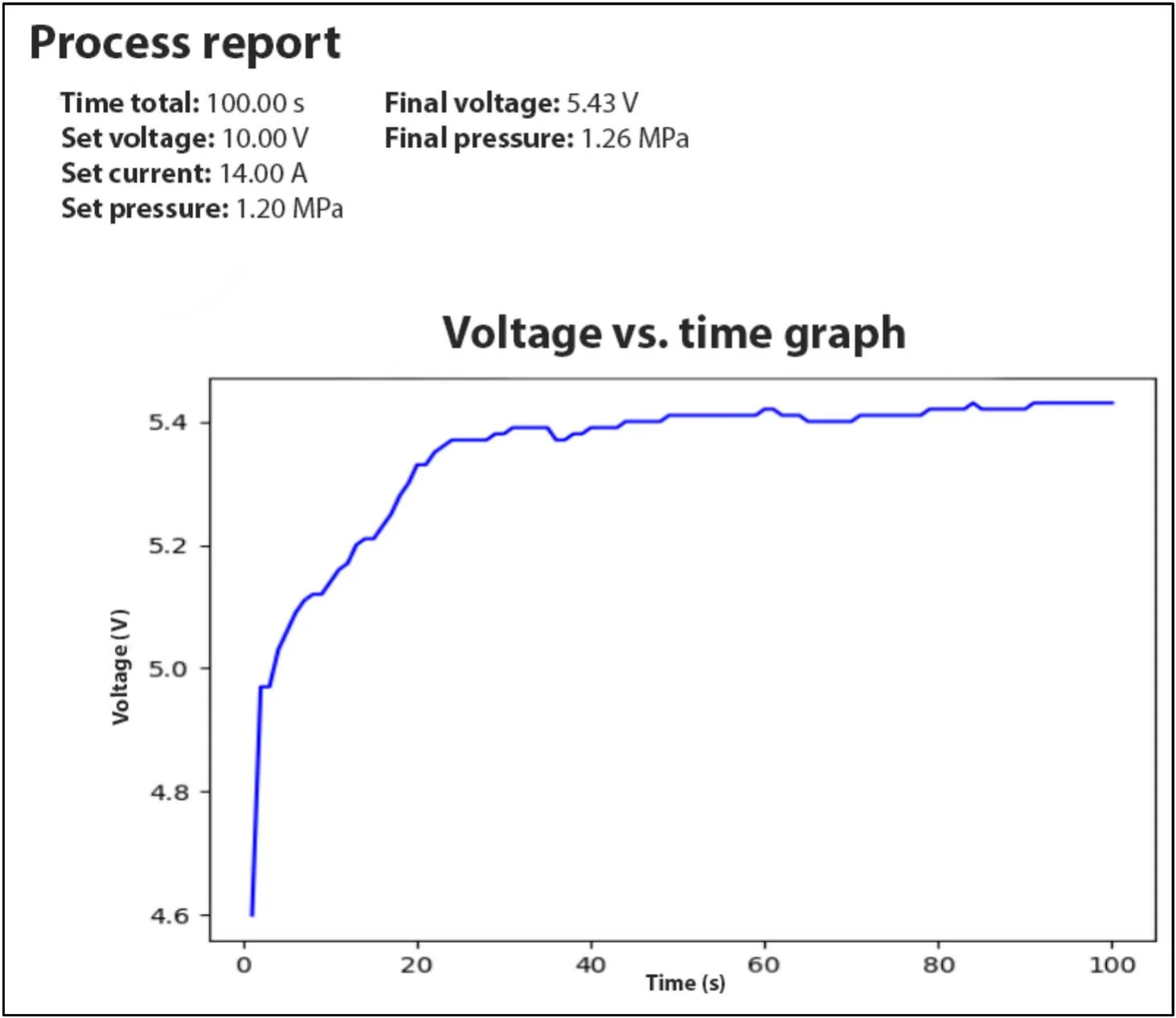
PDF report automatically generated by the software, displaying user-defined welding parameters and the corresponding voltage–time profile.

During welding, the custom software logged process variables at 1 Hz and exported detailed data in.txt format (Fig. 10), enabling quantitative analysis of the weld cycle. The software automatically calculates key parameters relevant to resistance welding of composites. For the GF/PEI validation set, average power was 45.69 ± 1.1 W, average power density 0.14 ± 0.03 W/mm^2^, and average electrical resistivity 0.23 ± 0.02 Ω. In addition, the system generates a comprehensive PDF report containing the final user-defined parameters and a voltage-versus-time graph (Fig. 11).

Post-weld performance was further characterized by lap shear strength (LSS) testing and optical microscopy. LSS tests were conducted on a Shimadzu AG-X universal testing machine (50 kN load cell) following ASTM D5868 at a constant crosshead speed of 1.5 mm/min until failure. The mean LSS obtained for the GF/PEI joints was 29.83 ± 1.2 MPa, demonstrating repeatable and structurally sound consolidation.

For microstructural analysis, welded specimens were embedded in epoxy resin (2:1 resin-to-hardener ratio) and cured for 24 h in silicone molds. After curing, samples were prepared by sequential grinding (grit sizes 100 through 2000) followed by polishing with 3 µm diamond paste. Fractographic surfaces from LSS-tested specimens were examined in the as-fractured state to preserve native fracture features. Microscopy was performed with a Zeiss Stemi 2000 stereomicroscope, a Nikon Epiphot 200 microscope, and a motorized Zeiss AxioImager Z2m at the Materials Imaging Laboratory (LaiMat), Department of Materials and Technology, School of Engineering and Science, Guaratinguetá Campus.

**Fig. 12:**
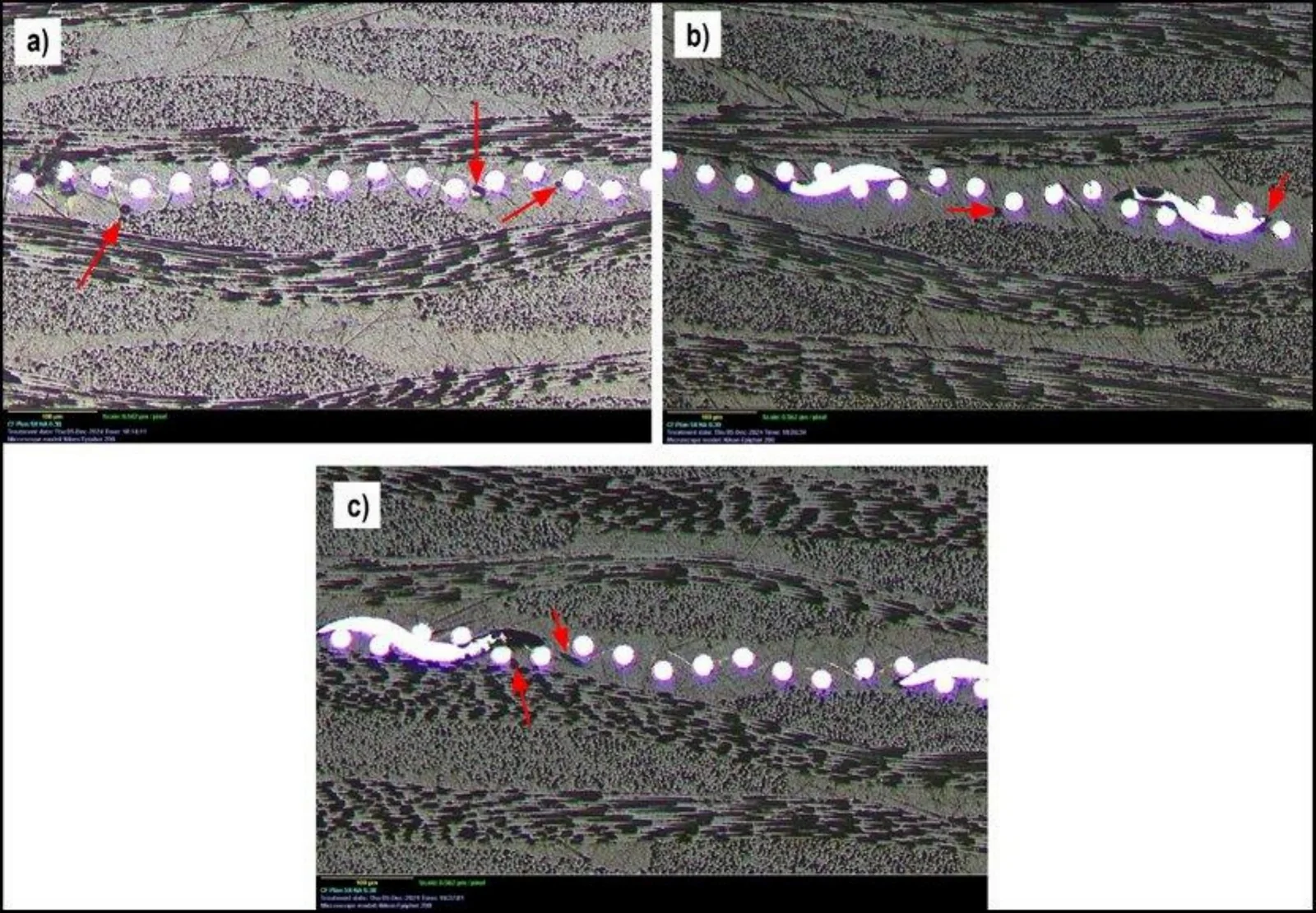
Optical micrograph of the central region of a welded joint showing low void content (arrows indicate pores/voids): a) region with an essentially planar weld line and evenly spaced mesh wires; b) region with the most pronounced mesh undulation; c) region with the most pronounced mesh undulation.

**Fig. 13:**
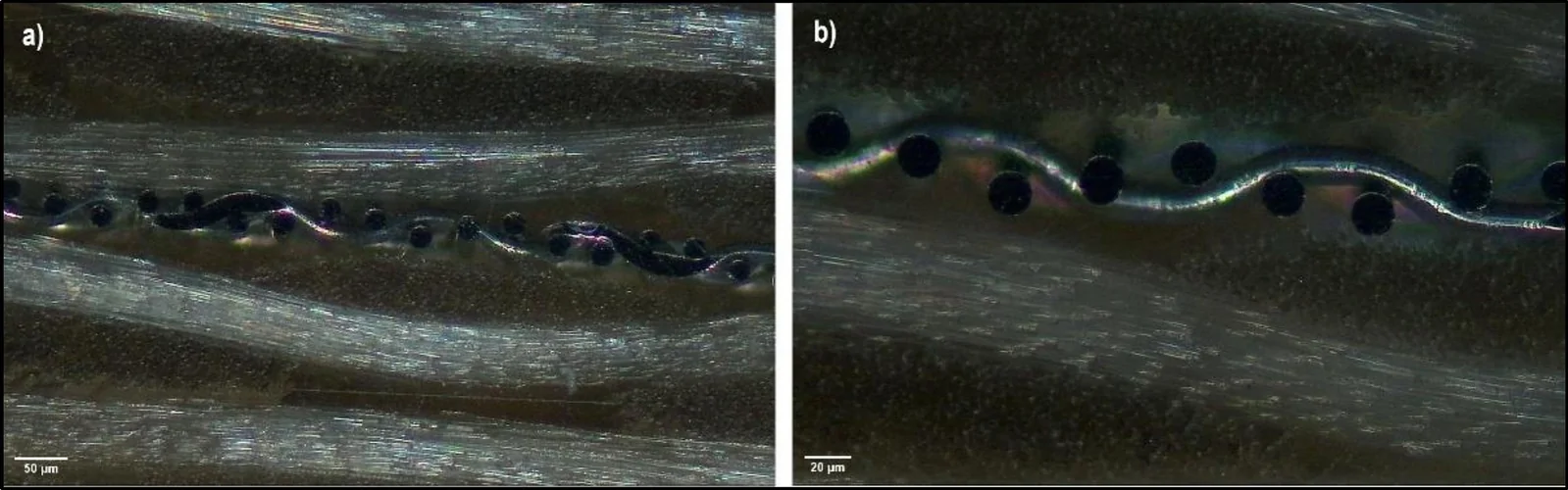
Dark-field optical micrograph demonstrating fiber integrity and uniform matrix encapsulation of the reinforcing mesh after welding: a) Overview of the joint at lower magnification (50 µm) and b) Detail of the same interface at higher magnification (20 µm).

Optical micrographs of the central joint region (Fig. 12 a-c) revealed good interfacial consolidation with only minor porosity and voids (arrows). These minor defects are attributed to residual solvent in the PEI matrix or slight thermal gradients during resistive heating. Dark-field imaging (Fig. 13 a,b) showed no delamination or damage to longitudinally oriented fibers. The reinforcing meshes were well encapsulated by the matrix, indicating strong fiber–matrix interaction and effective consolidation.

### 7.1 Capabilities and limitations of the hardware and software

The resistance welding system and associated software were validated for the specific application of joining GF/PEI thermoplastic composites. The following bulleted list summarizes the demonstrated capabilities together with current limitations:

• **Real-time process monitoring and data logging:** Voltage, current, and derived metrics (power, power density, resistivity) are acquired at 1 Hz and automatically exported to.txt files and PDF reports containing time-series graphs.
• **Data integrity and error handling:** serial communication operates under bounded timeouts; each frame received from the microcontroller is validated for structure and numeric content before acceptance, and malformed frames are discarded without interrupting acquisition; responses from the power supply are validated against the expected protocol echo prior to parsing. Each logged record carries a timestamp measured from the system clock at the instant of acquisition rather than an incremented counter, and the.txt log and PDF report are generated from the same in-memory arrays, ensuring consistency between outputs.
• **Repeatability and precision:** Low variability across replicates (power ± 1.1 W; LSS coefficient of variation ≈ 4%) under fixed operating conditions (10 V, 14 A, 1 MPa, 100 s), confirming stable and reproducible performance.
• **Joint mechanical performance:** Achieved mean lap shear strength of 29.83 ± 1.2 MPa with minimal void content, suitable for structural applications of GF/PEI composites.
• **Microstructural quality assessment:** Integrated protocols enable semiquantitative evaluation of consolidation (void content, fiber–matrix interaction) via optical microscopy and fractography, confirming effective welding without delamination or fiber damage.
• **Compliance with standards:** Fully compatible with ASTM D5868 for both welding and mechanical testing; sensor calibration procedures established for voltage, current, and temperature (50–400 °C).
• **Material and geometry specificity:** Validation performed on one thermoplastic composite system (GF/PEI) and one joint geometry; performance on other matrices, fiber types, thicknesses, or joint configurations requires further parametric characterization.
• **Temperature range:** Thermocouple-based monitoring is limited to 50–400 °C; higher-temperature applications would require different sensors or non-contact methods (e.g., infrared thermography).
• Thermal characterization of the support structure: direct measurement of the temperature gradient across the wooden base during welding was not performed; such characterization is recommended for applications involving interface temperatures or cycle durations beyond those validated here (GF/PEI, 100 s).
• **Assumptions in derived metrics:** Power density and resistivity calculations assume uniform current distribution across the joint area; edge effects or non-uniform heating in larger/complex geometries may reduce accuracy.
• **Software functionality:** Current version provides robust logging and PDF reporting but lacks advanced features such as closed-loop feedback control, real-time statistical process control, or direct integration with external databases/machine-learning pipelines.
• **Communication robustness:** the serial protocol does not implement checksum or cyclic-redundancy verification, relying on structural and numeric validation of each frame; link-loss watchdog and automatic reconnection routines are not implemented, and interruption of the serial connection during a run requires operator intervention.
• **Parameter space coverage:** Performance demonstrated at a single optimized parameter set; systematic mapping across wider ranges of voltage, current, pressure, and time is still in progress.

Overall, the hardware and software platform has been successfully validated for resistance welding of GF/PEI composites, delivering repeatable joints with high mechanical performance and good microstructural quality. Ongoing work is extending the operational envelope and software capabilities to broader composite joining applications.

### CRediT authorship contribution statement

**Jonas Frank Reis:** Writing – review & editing, Writing – original draft, Visualization, Validation, Software, Project administration, Methodology, Investigation, Funding acquisition, Formal analysis, Data curation, Conceptualization. **Luís Felipe Barbosa Marques:** Writing – review & editing, Visualization, Validation, Project administration, Methodology, Investigation. **Miguel Omena Lucas Vieira:** Writing – review & editing, Software, Resources, Investigation, Data curation. **Davi Nascimento Gomes:** Writing – review & editing, Software, Investigation. **Thiago Alves Kotz:** Writing – review & editing, Methodology. **Luis Rogerio de Oliveira Hein:** Visualization, Validation, Supervision, Resources, Funding acquisition, Formal analysis, Conceptualization.

## Declaration of competing interest

The authors declare that they have no known competing financial interests or personal relationships that could have appeared to influence the work reported in this paper.

## References

[1] Talreja R., Waas A.M. (2022). Concepts and definitions related to mechanical behavior of fiber reinforced composite materials. Compos. Sci. Technol..

[2] Sharma H., Kumar A., Rana S., Sahoo N.G., Jamil M., Kumar R., Sharma S., Li C., Kumar A., Eldin S.M., Abbas M. (2023). Critical review on advancements on the fiber-reinforced composites: Role of fiber/matrix modification on the performance of the fibrous composites. J. Mater. Res. Technol..

[3] Marques L.F.B., Reis J.F., dos Santos T.S.R., Lucas R.R., Sales-Contini R. de C.M., Mota R.P., Botelho E.C., Hein L.R. de O. (2026). Surface treatments for polymer composite welding: a systematic review. J. Thermoplast. Compos. Mater..

[4] Rohart V., Laberge Lebel L., Dubé M. (2020). Improved adhesion between stainless steel heating element and PPS polymer in resistance welding of thermoplastic composites. Compos. B Eng..

[5] Mariam M., Afendi M., Abdul Majid M.S., Ridzuan M.J.M., Gibson A.G. (2018). Tensile and fatigue properties of single lap joints of aluminium alloy/glass fibre reinforced composites fabricated with different joining methods. Compos. Struct..

[6] Botelho E.C., Pardini L.C., Rezende M.C. (2007). Evaluation of hygrothermal effects on the shear properties of Carall composites. Mater. Sci. Eng. A.

[7] Frank Reis J., Cintra I.L.R., Marques L.F.B., Ferrandini P.L., Abrahao A.B.M., Lima M.S.F., Botelho E.C. (2024). Study on YB-laser welding applied on aluminum/polymer composites. J. Adhes. Sci. Technol..

[8] Farahani R.D., Dubé M. (2017). Novel heating elements for induction welding of carbon fiber/polyphenylene sulfide thermoplastic composites. Adv. Eng. Mater..

[9] Villegas I.F., Bersee H.E.N. (2010). Ultrasonic welding of advanced thermoplastic composites: an investigation on energy-directing surfaces. Adv. Polym. Tech..

[10] Bhudolia S.K., Perrotey P., Joshi S.C. (2017). Enhanced vibration damping and dynamic mechanical characteristics of composites with novel pseudo-thermoset matrix system. Compos. Struct..

[11] Boria S., Scattina A., Belingardi G. (2017). Impact behavior of a fully thermoplastic composite. Compos. Struct..

[12] Matadi Boumbimba R., Coulibaly M., Khabouchi A., Kinvi-Dossou G., Bonfoh N., Gerard P. (2017). Glass fibres reinforced acrylic thermoplastic resin-based tri-block copolymers composites: Low velocity impact response at various temperatures. Compos. Struct..

[13] Bhudolia S.K., Joshi S.C. (2018). Low-velocity impact response of carbon fibre composites with novel liquid Methylmethacrylate thermoplastic matrix. Compos. Struct..

[14] Obande W., Ray D., Brádaigh C.M.Ó. (2019). Viscoelastic and drop-weight impact properties of an acrylic-matrix composite and a conventional thermoset composite – a comparative study. Mater. Lett..

[15] Kinvi-Dossou G., Matadi Boumbimba R., Bonfoh N., Garzon-Hernandez S., Garcia-Gonzalez D., Gerard P., Arias A. (2019). Innovative acrylic thermoplastic composites versus conventional composites: improving the impact performances. Compos. Struct..

[16] Barbosa L.C.M., Bortoluzzi D.B., Ancelotti A.C. (2019). Analysis of fracture toughness in mode II and fractographic study of composites based on Elium® 150 thermoplastic matrix. Compos. B Eng..

[17] Shanmugam L., Kazemi M.E., Rao Z., Lu D., Wang X., Wang B., Yang L., Yang J. (2019). Enhanced Mode I fracture toughness of UHMWPE fabric/thermoplastic laminates with combined surface treatments of polydopamine and functionalized carbon nanotubes. Compos. B Eng..

[18] Kropka M., Muehlbacher M., Neumeyer T., Altstaedt V. (2017). From UD-tape to final Part – a comprehensive approach towards thermoplastic composites. Procedia CIRP.

[19] Yang Y., Boom R., Irion B., van Heerden D.J., Kuiper P., de Wit H. (2012). Recycling of composite materials. Chem. Eng. Process..

[20] Kazemi M.E., Shanmugam L., Lu D., Wang X., Wang B., Yang J. (2019). Mechanical properties and failure modes of hybrid fiber reinforced polymer composites with a novel liquid thermoplastic resin, Elium®. Compos. Part A Appl. Sci. Manuf..

[21] Bhudolia S.K., Joshi S.C., Bert A., Yi Di B., Makam R., Gohel G. (2019). Flexural characteristics of novel carbon methylmethacrylate composites. Compos. Commun..

[22] Brassard D., Dubé M., Tavares J.R. (2021). Modelling resistance welding of thermoplastic composites with a nanocomposite heating element. J. Compos. Mater..

[23] Zhao T., Palardy G., Villegas I.F., Rans C., Martinez M., Benedictus R. (2017). Mechanical behaviour of thermoplastic composites spot-welded and mechanically fastened joints: a preliminary comparison. Compos. B Eng..

[24] Liu S., Wang Y., Fan T., Wu Z., Li Y. (2024). The influence of process parameters and carbon nanotubes on composite material joints. Polym. Compos..

[25] Heathman N., Koirala P., Yap T., Emami A., Tehrani M. (2023). In situ consolidation of carbon fiber PAEK via laser-assisted automated fiber placement. Compos. B Eng..

[26] Zhao D., Zhang Q., Fan X., Zhao S. (2018). Review on joining process of carbon fiber-reinforced polymer and metal: methods and joining process. Rare Metal Mater. Eng..

[27] Absi C., Alsinani N., Laberge Lebel L. (2022). Carbon fiber reinforced poly(ether ether ketone) rivets for fastening composite structures. Compos. Struct..

[28] Chen C., Zhang W., Li Y., Gu L., Luo Y., Qin M., Zhang J., He Z., Wang H., Zheng Y., Yao D. (2023). The influence of moisture absorption-drying of composite materials on the bonding performance of the joints. Macromol. Mater. Eng..

[29] Rakesh P.K., Kumar R., Dixit A., Gurjar S.S., Kumar J. (2024). Effect of mechanical fastening versus adhesively bonded joints of polymer composites on lap joint strength. J. Adv. Manuf. Syst..

[30] Yan D., Shan Z., Zang Y., Yang H. (2023). Study on interlayer bonding properties of multi-material composite cast mold. Mater. Lett..

[31] Xiong X., Wang D., Ren R., Zhao P., Cui X., Ji S. (2021). Improved mechanical properties of resistance welded joints of thermoplastic composites by versatile graphene oxide. J. Adhes. Sci. Technol..

[32] Goto K., Imai K., Arai M., Ishikawa T. (2019). Shear and tensile joint strengths of carbon fiber-reinforced thermoplastics using ultrasonic welding. Compos. A.

[33] Haque R. (2018). Quality of self-piercing riveting (SPR) joints from cross-sectional perspective: a review. Arch. Civ. Mech. Eng..

[34] Xiong X., Zhao P., Ren R., Zhang Z., Cui X., Ji S. (2020). Enhanced resistance-welding hybrid joints of titanium alloy/thermoplastic composites using a carbon-nanotube lamina. Diam. Relat. Mater..

[35] Dey K., Gobetti A., Ramorino G. (2023). Advances in understanding of multiple factors affecting vibration weld strength of thermoplastic polymers. J. Adv. Join. Process..

[36] Paranjpe N., Uddin M.N., Rahman A.S., Asmatulu R. (2024). Effects of surface treatment on adhesive performance of composite-to-composite and composite-to-metal joints. Processes.

[37] Abdel Wahab M.M. (2012). Fatigue in adhesively bonded joints: a review. ISRN Mater. Sci..

[38] Hossein Abadi R., Refah Torun A., Mohammadali Zadeh Fard A., Choupani N. (2020). Fracture characteristics of mixed-mode toughness of dissimilar adherends (cohesive and interfacial fracture). J. Adhes. Sci. Technol..

[39] Xiong X., Wang D., Wei J., Zhao P., Ren R., Dong J., Cui X. (2021). Resistance welding technology of fiber reinforced polymer composites: a review. J. Adhes. Sci. Technol..

[40] Bhudolia S.K., Gohel G., Leong K.F., Islam A. (2020). Advances in ultrasonicwelding of thermoplastic composites: a review. Materials.

[41] Li X., Zhang T., Li S., Liu H., Zhao Y., Wang K. (2021). The effect of cooling rate on resistance-welded CF/PEEK joints. J. Mater. Res. Technol..

[42] Zhao P., Zhang Z., Li Y., Tian L., Wang C., Xiong X. (2022). Resistance welding of thermoplastic composites via a novel carbon nanofilm implant. Mater. Lett..

[43] Zhao G., Li M., Zhao Y., Zhou X., Yu H., Jian X., Zhang S., Xu J. (2024). Rotating gliding arc plasma: innovative treatment for adhesion improvement between stainless steel heating elements and thermoplastics in resistance welding of composites. Compos. B Eng..

[44] B. Jiang, Q. Chen, J. Yang, Advances in joining technology of carbon fiber-reinforced thermoplastic composite materials and aluminum alloys, (n.d.). https://doi.org/10.1007/s00170-020-06021-2/Published.

[45] Nino G.F., Ahmed T.J., Bersee H.E.N., Beukers A. (2009). Thermal NDI of resistance welded composite structures. Compos. B Eng..

[46] Wei D., Gu Y., Zhu H., Li M., Wang S. (2022). Influence of electrical heating metal mesh and power density on resistance welding of carbon fiber/PEEK Composite. Polymers (basel)..

[47] Zhang X., Chen Y., Hu J. (2018). Recent advances in the development of aerospace materials. Prog. Aerosp. Sci..

[48] Li W., Palardy G. (2023). Investigation of welding repair methods for thermoplastic composite joints. Compos. B Eng..

[49] Zhao G., Chen T., Xia X., Zhao Y., Liu C., Jian X., Zhang S., Xu J. (2023). Resistance welding of thermoplastic composites with nanofiber films prepared by electrospinning technique. Compos. B Eng..

[50] Marques L.F.B., Reis J.F., Callisaya E.S., de Paula Kodaira F.V., Botelho E.C., Mota R.P., de Oliveira Hein L.R. (2026). Enhancing the resistance welding of GF/PEI composites via a PECVD-deposited HMDSO coating on a heating element. ACS Omega.

[51] Totla H.S., Gupta A., Mishra S., Sharma S., Selvaraj S.K. (2023). Resistance welding analysis of thermoplastic composite structures in aeronautical applications. Int. J. Interact. Des. Manuf..

[52] Villegas I.F., Moser L., Yousefpour A., Mitschang P., Bersee H.E.N. (2013). Process and performance evaluation of ultrasonic, induction and resistance welding of advanced thermoplastic composites. J. Thermoplast. Compos. Mater..

[53] Russello M., Catalanotti G., Hawkins S.C., Falzon B.G. (2020). Welding of thermoplastics by means of carbon-nanotube web. Compos. Commun..

[54] Reis J.F., Abrahao A.B.M., Costa M.L., Botelho E.C. (2018). Assessment of the interlaminar strength of resistance-welded PEI/carbon fibre composite. Weld. Int..

[55] Stankiewicz K., Lipkowski A., Kowalczyk P., Giżyński M., Waśniewski B. (2024). Resistance welding of thermoplastic composites, including welding to thermosets and metals: a review. Materials.

[56] Brassard D., Dubé M., Tavares J.R. (2019). Resistance welding of thermoplastic composites with a nanocomposite heating element. Compos. B Eng..

[57] Pramanik A., Basak A.K., Dong Y., Sarker P.K., Uddin M.S., Littlefair G., Dixit A.R., Chattopadhyaya S. (2017). Joining of carbon fibre reinforced polymer (CFRP) composites and aluminium alloys – a review. Compos. Part A Appl. Sci. Manuf..

[58] Larsen L., Endrass M., Jarka S., Bauer S., Janek M. (2025). Exploring ultrasonic and resistance welding for thermoplastic composite structures: Process development and application potential. Compos. B Eng..

[59] Shi H., Villegas I.F., Bersee H.E.N. (2013). Strength and failure modes in resistance welded thermoplastic composite joints: effect of fibre-matrix adhesion and fibre orientation. Compos. Part A Appl. Sci. Manuf..

[60] Long W., Zhou X., Du B., Cheng X., Su G., Chen L. (2024). Enhancing the lap shear performance of resistance-welded GF/PP thermoplastic composite by modifying metal heating elements with silane coupling agent. Materials.

[61] Dubé M., Hubert P., Gallet J.N.A.H., Stavrov D., Bersee H.E.N., Yousefpour A. (2012). Metal mesh heating element size effect in resistance welding of thermoplastic composites. J. Compos. Mater..

